# T_1_ and T_2_ measurements of the neonatal brain at 7 T

**DOI:** 10.1002/mrm.30403

**Published:** 2024-12-13

**Authors:** Aiman Mahmoud, Raphael Tomi-Tricot, David Leitão, Philippa Bridgen, Anthony N. Price, Alena Uus, Arnaud Boutillon, Andrew J. Lawrence, Daniel Cromb, Paul Cawley, Maria Deprez, Enrico De Vita, Sharon L. Giles, Mary A. Rutherford, A. David Edwards, Joseph V. Hajnal, Tomoki Arichi, Shaihan J. Malik

**Affiliations:** 1 Imaging Physics and Engineering Research Department, School of Biomedical Engineering and Imaging Sciences, King’s College London, London, UK; 2 London Collaborative Ultrahigh field System (LoCUS), King’s College London, London, UK; 3 MR Research Collaborations, Siemens Healthcare Limited, Frimley, UK; 4 Guy’s and St. Thomas’ NHS Foundation Trust, London, UK; 5 Early Life Imaging Research Department, School of Biomedical Engineering and Imaging Sciences, King’s College London, London, UK; 6 Department of Psychological Medicine, Institute of Psychiatry, Psychology, and Neuroscience, King’s College London, London, UK; 7 Center for Neurodevelopmental Disorders, King’s College London, London, UK

**Keywords:** neonatal imaging, relaxation times, ultrahigh field

## Abstract

**Purpose:**

To determine the expected range of NMR relaxation times (T_1_ and T_2_) in the neonatal brain at 7 T.

**Methods:**

Data were acquired in a total of 40 examinations on infants in natural sleep. The cohort included 34 unique subjects with postmenstrual age range between 33 and 52 weeks and contained a mix of healthy individuals and those with clinical concerns. Single-slice T_1_ and T_2_ mapping protocols were used to provide measurements in white matter, cortex, cerebellum, and deep gray matter. Automatic image segmentation of a separate T_2_-weighted brain volume was used to define regions of interest for analysis.

**Results:**

Linear regression was used to estimate relaxation times at term equivalent age (40 weeks postmenstrual age). $T_{1}^{40wk}$ with 95% confidence intervals was measured to be 2933 [2893, 2972] ms in white matter; 2653 [2604, 2701] ms in cerebellum; and 2486 [2439, 2532] ms in basal ganglia. $T_{2}^{40wk}$ was estimated as 119 [116, 121] ms in white matter, 99 [96, 102] ms in cerebellum, and 90 [89, 92] ms in basal ganglia. Most tissue-relaxation times showed a significant negative correlation with postmenstrual age, with the strongest correlation seen in cerebellum.

**Conclusions:**

We describe neonatal brain tissue and age-specific T_1_ and T_2_ relaxation values at 7 T. The presented values differ substantially from both adult values at 7 T and neonate values measured at lower field strengths, and will be essential for pulse-sequence optimization for neonatal studies.

## 1 Introduction

Ultrahigh-field (UHF) MR scanners offer increased signal-to-noise ratio and enhanced susceptibility contrast.^1,2^ Neonatal MR brain imaging MRI could particularly benefit from this technology, given the need for high resolution due to the smaller size of the developing brain. Recent work has demonstrated the possible gains in image quality that can be obtained from UHF MRI in neonates but have so far used largely unoptimized sequences.^3,4^ A key challenge for optimizing sequences for the neonatal population at 7 T is the lack of data on tissue-relaxation properties at this field strength. Both T_1_ and T_2_ relaxation times change dramatically during development^5^ and are expected to be substantially longer in neonates compared with adults due to the increased water content of immature brain tissue. Furthermore, T_1_ values are known to depend on the static field strength^6^ (as demonstrated in previous neonatal studies at 3 T^7–9^ and recently at ultralow field [64 mT]^10^) and thus would be expected to generally be longer at UHF. T_2_ values are expected to be independent of field strength (but can also be lower due to diffusion effects^11^).

This work presents the first systematic characterization of T_1_ and T_2_ relaxation times in human neonates on a 7T MR system.

## 2 Methods

A cohort of 34 subjects (22 male) were included in this study over a total of 40 exams (median gestational age at birth [GA] 36^+0^ weeks, range 27^+6^–42^+1^ weeks; median postmenstrual age [PMA] at scan: 39^+5^ weeks, range 33^+4^–52^+6^ weeks) on a 7 T system (MAGNETOM Terra; Siemens Healthcare) at the LoCUS MRI Unit, St. Thomas’ Hospital, London, following written parental consent (NHS REC: 19/LO/1384). The cohort included both healthy individuals and those with various clinical indications for brain imaging; full details are given in Table S1.

### 2.1 Acquisition

A 1-transmit/32-receive head coil (Nova Medical, Wilmington, MA, USA) was used with a locally modified safety model enforcing conservative operating limits, defined following a neonatal-specific risk assessment.^3,12^ Physiological vital signs (heart rate, oxygen saturation, and temperature) were monitored by a Philips-Invivo Expression MR400 monitor and reviewed throughout the scan by clinical staff. Hearing protection was provided using dental putty molds in the external auditory meatus (President Putty; Coltene Whaledent, Mahwah, NJ, USA) and using inflatable positioning pads (Pearltec, Zurich CH). Infants were scanned in natural sleep following feeding.

**FIGURE 1:**
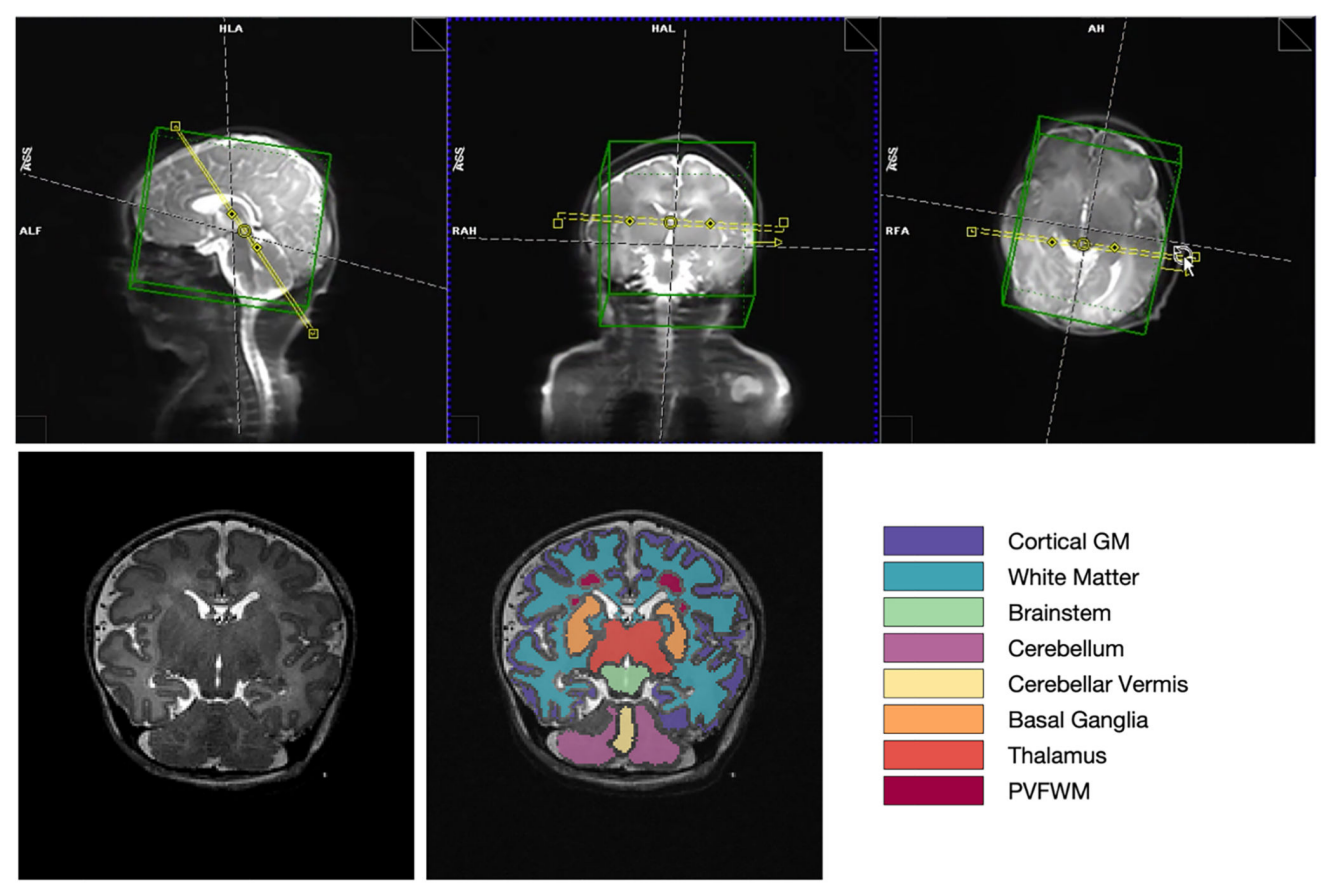
*Top row*: Example of planning single oblique slice for relaxometry. *Left*: Localizer image showing the planned slice (*yellow*) in three planes (*green box is the volume used for shimming*). *Bottom row*: Left: example T_2_-weighted image from this oblique slice. Right = T_2_w image overlaid with example image segmentation from this subject, used for region of interest analysis (details of segmentation methods are given in section 2.5). GM, gray matter; PVFWM, periventricular frontal white matter.

The objective of this study was to obtain information on relaxation times for a range of tissue types and to examine variation with maturation across the neonatal period, so that these values could be used to further optimize sequence parameters for 7T population–specific acquisition. This study was part of a broader preliminary investigation of neonatal MRI at 7 T^3^; thus, to increase efficiency, a protocol was established using a single oblique coronal slice angulated to pass through multiple tissues of interest (white matter, cortex, deep gray matter, and cerebellum). Figure 1 shows an example localizer image used for planning this slice. Planning of the acquisition slice was performed by an experienced radiographer and a pediatric neurologist for consistency across subjects.

### 2.2 T_1_ mapping

For T_1_ mapping, we used a single-shot turbo spin echo (TSE) acquisition with adiabatic inversion-recovery preparation, using variable delay times (Ti=0.5, 1.0, 1.5, 2.0, 3.0. 4.0, and 5.0 s plus no inversion) and the following parameters: 0.8 × 0.8 mm in-plane with slice thickness 1.6 mm, GRAPPA factor = 2 and 5/8 partial Fourier sampling, echo time (TE) = 77 ms, repetition time (TR) = 10 s, and refocusing flip angle = 120°. A variable delay was used between acquisitions to ensure consistent magnetization recovery, such that *TR* − (*T*_*i*_ + *T*_shot_) + *T*_delay_ ≥ 20*s*; *T*_shot_ is the TSE shot duration (~370 ms), and *T*_delay_ is the added delay. A total of eight images were obtained for each subject, with acquisition time of 1 min 40 s including delays.

The signal equation for this sequence is as follows:

(1) $$S\left(T_{i}\right)=\left|S_{0}\left\{1-2(1-\epsilon )\text{exp}\left(-\frac{T_{i}}{T_{1}}\right)\right\}\right|$$

where S_0_ is a term including factors related to the TSE readout (common to all images); *T*_*i*_ is the inversion delay time; and *ϵ* is the inversion inefficiency. For perfect inversion, we would expect *ϵ* = 0; however, this same term can also be used to compensate for magnetization transfer–induced bi-exponential longitudinal relaxation observed at UHF.^13,14^ The *ϵ* term allows *S*(*T*_*i*_ → 0) < *S*(*T*_*i*_ → ∞), which is the case when there is an initial period of fast recovery^15^ (*S*(*T*_*i*_ → ∞) denotes the signal obtained in the image with no inversion pulse). All data analysis used *MATLAB* R2023a (The MathWorks, Natick, MA, USA); magnitude images were fit to Eq.(1) using least squares fitting via *MATLAB* function *fmincon*. Fitting was constrained with the following bounds: 0 < *S*_0_ < 1.3 × *S*(*T*_*i*_ → ∞) and 0 < *T*_1_ < 6*s*,0 < *ϵ* < 0.5.

Before T_1_ estimation, image registration was used to correct for small amounts of motion between acquisitions (using *MATLAB* function *imregister*) with rigid-body transformation only (translation and rotation). This procedure cannot compensate for through-plane motion; therefore, in cases in which registration proved unsuccessful, images from individual *T*_*i*_s were excluded manually after visual inspection (see Table S1 for details).

### 2.3 T_2_ mapping

The T_2_ mapping protocol used a multishot two-dimensional (2D) TSE acquisition with equivalent resolution, slice thickness, field of view, and GRAPPA factor to the T_1_ mapping sequence. The TSE echo train length of 24, with spacing 11.2ms, was divided into three k-spaces with nominal TEs of 59, 154, and 283 ms (corresponding to the 5th, 13th, and 24th echoes, respectively). The refocusing flip angle was set at 180°, with TR of 5 s, resulting in a total scan duration of 1 min 10 s. In addition, a three-dimensional (3D) ${\text{B}}_{1}^{+}$ map was obtained using actual flip-angle imaging^16^ (resolution = 2.2 × 2.2 × 3 mm^3^; 5/8 partial Fourier and GRAPPA factor = 3; TR = 23.5/117.5 ms; nominal flip angle *θ*_nominal_ = 60°). Relative ,${\text{B}}_{1}^{+}$ was obtained by dividing the flip angle from the actual flip-angle imaging map by the nominal value (i.e., $B_{1}^{rel\;}=\frac{\theta_{AFI}}{\theta_{\text{nominal}\;}}$).

The signal obtained from the 2D-TSE sequence is dependent on slice profile and $B_{1}^{rel\;}$. We used a dictionary-based reconstruction similar to Ben-Eliezer et al.^17^ to reconstruct T_2_ correcting for these effects. Briefly, this method requires a Bloch simulation of the pulse sequence using different relaxation times and $B_{1}^{rel\;}$ values to construct a dictionary of signal evolutions, which is then used to infer T_2_.

Bloch simulations were performed for one entire echo train from the sequence (including all gradient wave-forms), using a grid of isochromats covering a voxel of size of 1 mm in the readout direction and 4 mm through-slice with 51 and 501 isochromats, respectively, in each direction (i.e., a total of 25 551). Signal was predicted by integrating transverse magnetization over the whole voxel and extracting the magnitude at each TE. This was repeated for 32 different T_2_ values (equally spaced between 10 and 480 ms), 24 different T_1_ values (500 to 4000 ms), and 16 different values of $B_{1}^{rel\;}$ (0.2 to 1.5). A dictionary was created by considering signal only at the 5th, 13th, and 24th echoes corresponding to centers of k-space. The dictionary was interpolated to a higher resolution of 1 ms in T_2_, 10 ms in T_1_, and 0.01 in $B_{1}^{rel\;}$ using linear interpolation. Interpolation of dictionaries has been described by others^18^ and appears reasonable in this case, given the smooth dependence on parameters (see Figure S1).

T_2_ estimation was performed by finding the maximum inner product between the dictionary and acquired signals. Unlike Ben-Eliezer et al., we did not estimate $B_{1}^{rel\;}$ from the TSE data (this was found to be unreliable in initial tests); rather, the measured $B_{1}^{rel\;}$ was used to identify the relevant subset of the dictionary, which was then used to match to the TSE data to estimate T_2_. The dictionary is also a weak function of T_1_, although this dependence is often ignored.^17^ The bias caused by using a fixed T_1_ was estimated by randomly sampling 200 000 entries from the full dictionary, then estimating T_2_ assuming T_1_ = 2.6 s (later shown to be appropriate for infants). Figure S2 shows that the bias remains less than 2% for parameter combinations of interest.

As infants were scanned in natural sleep, in some cases examinations were not completed. In eight examinations, the actual flip-angle imaging sequence was not obtained, and for these cases T_2_ was not estimated.

### 2.4 Phantom validation experiments

Inversion-recovery single-shot TSE is a robust T_1_ estimation method, with low sensitivity to $B_{1}^{+}$ inhomogeneity when using an adiabatic inversion pulse. The sequence was validated using a phantom (spherical flask filled with agarose gel); the experiment was performed once using normal settings and once after deliberately reducing the voltage of the inversion pulse from 129 to 80 V, to increase the impact of $B_{1}^{+}$ inhomogeneity on T_1_ measurement, to test the fitting approach.

The T_2_ estimation method was validated experimentally using the same phantom, against a 3D single spin-echo sequence with TR = 1 s and 2-mm isotropic resolution, giving an acquisition time of 5 min 27 s for one volume; five volumes were acquired with TEs = 50, 100, 150, 250, and 300 ms, resulting in a total acquisition time of 27 min 15 s. It was also compared with equivalent 3D spin-echo data acquired on 15-mL sample tubes filled with water doped with MnCl_2_ (concentrations of 0.01 and 0.05 mM).

### 2.5 Region-of-interest analysis

Image segmentation was used to automatically define regions of interest for calculating tissue averages of the T_1_ and T_2_ data. Segmentation was performed on separately acquired T_2_-weighted structural images acquired with 2D-TSE sequences (TE = 156 ms, acquired resolution = 0.6 mm, slice thickness = 1.2 mm) in at least two orthogonal planes, and an isotropic 3D volume created using slice-to-volume reconstruction.^3^ Segmentation used a combination of two neural network–based algorithms: An initial segmentation was generated using a brain extraction and parcellation algorithm developed for fetal imaging but optimized for neonate^19^; a second algorithm^20^ was then used to delineate the periventricular frontal white matter (PVFWM) region, which appears hyperintense in T_2_-weighted imaging in infants.^21^ Final tissue labels were generated by combining these labels in *MAT-LAB*, giving priority to PVFWM.

The single 2D slice of interest corresponding to the acquisition plane was interpolated from the 3D segmentations using the Medical Imaging Interaction Toolkit. Tissue labels were then eroded by 1 pixel using *MATLAB*’s built-in function *imerode* to reduce partial-volume effects in quantification. Linear regression was used to explore age-dependence of parameter values. Two analyses were run: the first considering the relationship between T_1_/T_2_ and PMA, and the second including also the postnatal age (PNA ≡ PMA − GA) to explore effect of premature birth. In both cases, mixed-effects models were used to account for repeat observations on the same infant with PMA/PNA treated as fixed effects and a random intercept related to subject id also included. Analysis used *MATLAB*’s *fitlme* function.

Perceptually uniform color maps taken from the “colorcet” package were used to display results in this work.^22^ Source code and data required to reproduce all presented figures are available online (see Data Availability Statement).

## 3 Results

### 3.1 Phantom validation experiments

Figure S3 shows the result of T_1_ mapping validation. No ${\text{B}}_{1}^{+}$-related inhomogeneity is visible in the maps when using the normal sequence; however, if the inversion-pulse voltage is reduced, the T_1_ map becomes very inhomogeneous if inversion inefficiency (*ϵ*) is not included in the fitting. Once *ϵ* is included, the method is demonstrated to be robust to ${\text{B}}_{1}^{+}$ variation.

Figure S4 compares the reference method for T_2_ mapping (3D spin echo) with the 2D approach used in this study; after using the dictionary-based reconstruction, the T_2_ values agreed (111 ± 5 ms for 2D method compared with 108 ± 3 ms from reference). Additional measurements were made on sample tubes containing MnCl_2_: The 2D method measured T_2_ of 246 ± 9 ms and 72 ± 2 ms for concentrations 0.01 and 0.05 mM, respectively, in agreement with corresponding reference measurements 227 ± 34 ms and 74 ± 13 ms.

### 3.2 In vivo parameter maps

**FIGURE 2:**
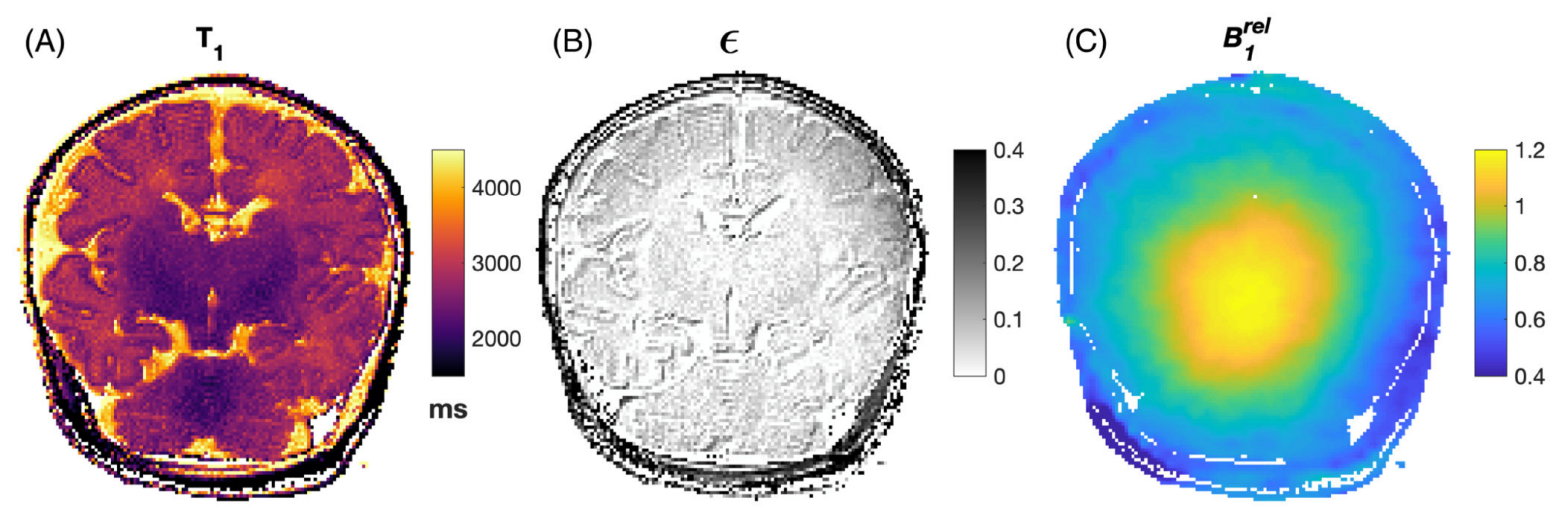
Results from T_1_ mapping on 1 subject (id#23; see Table S1). (A) Fitted T_1_ map. (B) Inversion inefficiency (*ϵ*) parameter. (C) Equivalent slice from relative B_1_ map.

**FIGURE 3:**
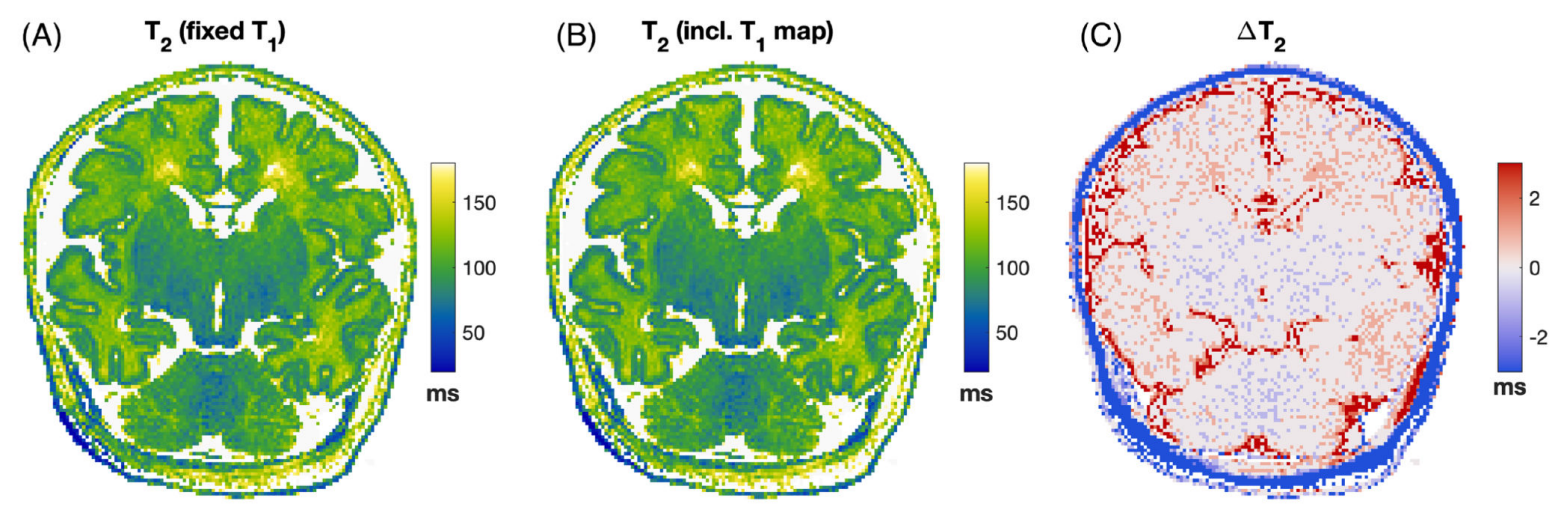
T_2_ estimation results from the same infant featured in Figure 2 (id#23). (A) T_2_ estimated using dictionary with fixed T_1_ = 2.6 s. (B) Dictionary estimation including voxel-wise T_1_ information. (C) Difference between these two estimates.

Figure 2 shows example results of T_1_ estimation for 1 subject (Subject 23; see Table S1) along with the same slice from $B_{1}^{rel\;}$ for reference. Figure 3 shows T_2_ estimation results from the same infant; Figure 3A is the result of the dictionary-based reconstruction assuming fixed T_1_ = 2.6 s, whereas Figure 3B shows the result of also including the voxel-wise T_1_ information (from Figure 2). The maps are very similar (mean absolute error is 0.73 ms and median absolute error is 0 ms); hence, all T_2_ maps were made using a dictionary with fixed T_1_ = 2.6 s to avoid the introduction of noise from the measured T_1_ map into T_2_ estimates. A collection of all fitted parameter maps is given in Figures S5–S7.

### 3.3 Whole cohort analysis

**FIGURE 4:**
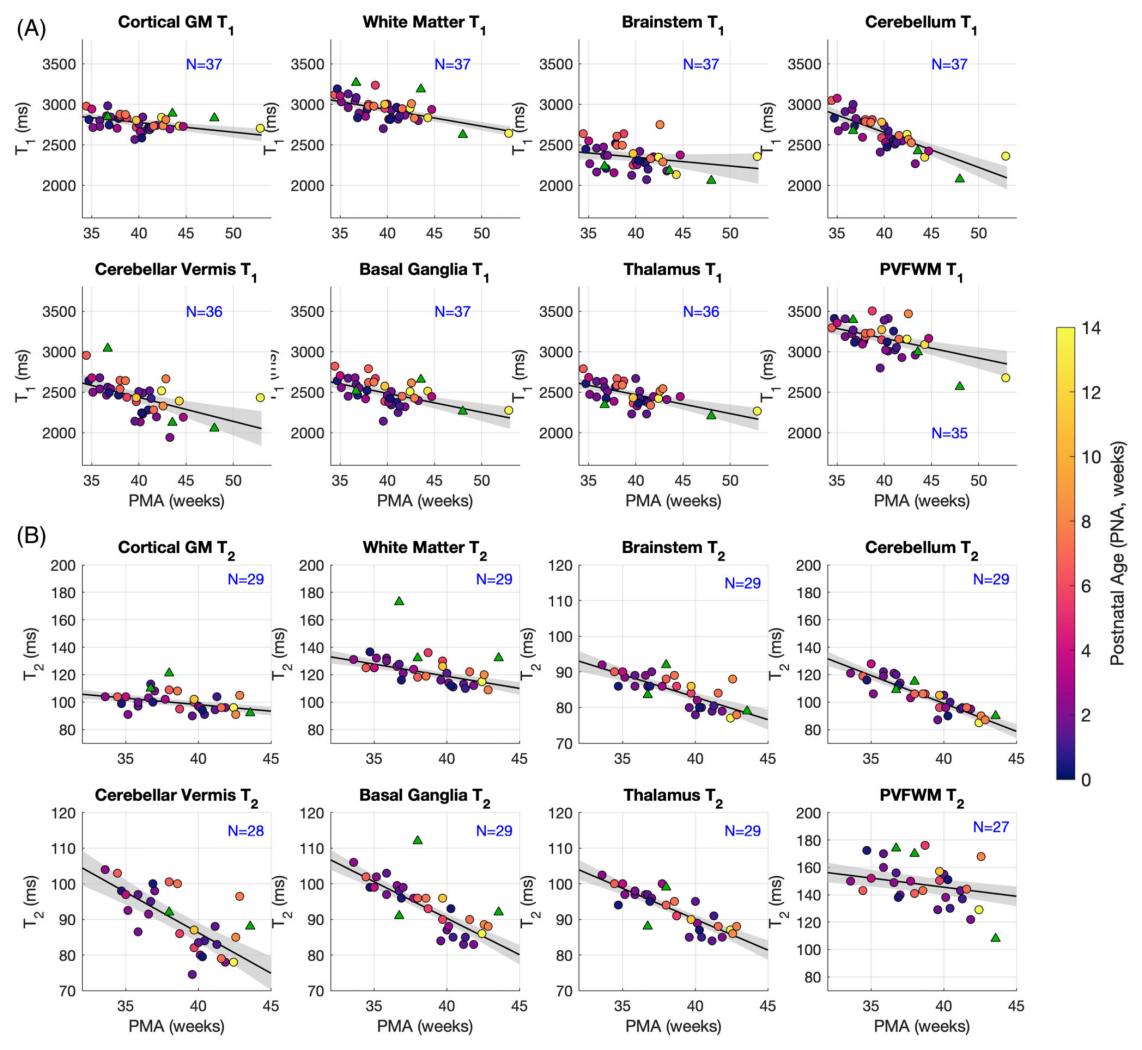
Region-of-interest measurements of relaxation parameters (regions of interest as defined by segmentation, illustrated in Figure 1). Top panels of (A) plot T_1_ and bottom panels of (B) give equivalent results for T_2_. Both parameters are plotted against postmenstrual age (PMA), with color representing postnatal age (PNA = PMA-GA). Linear trend line is superimposed (*solid line*) with 95% confidence interval (*shading*). The number of observations used for each regression (*N*) is indicated on each plot. *N* varies between anatomical regions, because some are not detected in the slice used for imaging in some subjects; there are also fewer subjects for T_2_ measurements due to missing $B_{1}^{rel}$. The samples indicated with triangles were excluded from regression (details in text). GA, gestational age; GM, gray matter; PNA, postnatal age; PVFWM, periventricular frontal white matter.

We successfully measured T_1_ in 40 and T_2_ in 32 exams. Figure 1 shows regions of interest obtained from image segmentation for the same infant depicted in Figures 2 and 3. Figure 4 shows the median T_1_ and T_2_ values within each of these regions of interest plotted against PMA, color-coded according to PNA. The result of linear regression is also marked on each plot. Three subjects deemed to have severe pathologies (cytomegalovirus infection, hypoxic ischemic encephalopathy, significant white-matter hyperintensity) are marked with triangles on the plots and were excluded from the analysis; an additional T_2_ data set was excluded due to severe motion artifacts. Table 1 summarizes results of single regression (considering only PMA). Both T_1_ and T_2_ were found to change significantly with PMA (*p* < 0.01) in all tissues with the exception of brainstem for T_1_. Rates of change with PMA are denoted as Δ_1,2_ in Table 1. Cerebellum shows particularly strong age dependence (Δ_1_ = −43[−53, −33] ms/week and Δ_2_ = −4.1[−5.0, −3.0] ms/week) with other tissues showing approximately half this effect. Results of analysis considering both PMA and PNA are found in Table S2.

## 4 Discussion

We describe reference values for tissue-relaxation times in human neonates at 7 T. One use of these is to act as a guide for future imaging protocol development for studies at UHF. Relaxation times in many of the tissues included in this study are age-dependent; therefore, we used linear regression to determine reference values for 40 weeks’ gestation and to quantify change over time.

As expected, the T_1_ values are longer than those previously described at lower field strengths; for example, at 3 T, Schneider et al. found $T_{1}^{40wk}=2077\pm 66\;\text{ms}$ for thalamus and approximately 2300 ms in cortex (depending on region) compared with 2469 [2428, 2510] ms and 2775 [2732, 2818] ms, respectively, in this study. Likewise, at the much lower field strength of 64 mT, Padormo et al. found $T_{1}^{40wk}=646\;\text{ms}$ in cerebellum and 628 ms in basal ganglia (compared with 2653 [2604, 2701] ms and 2486 [2439, 2532] ms) in this study. Fewer T_2_ measurements have been published in this age range, although Dong et al.^23^ reported $T_{2}^{40wk}\approx 250\;\text{ms}$ in white matter and 140 ms in thalamus measured at 3 T, both substantially longer than our measured values (119 [116, 121] ms and 90 [89, 91] ms, respectively).

Measured neonate values are, as expected, much longer than adult values measured at 7 T. For example, Rooney et al.^24^ measured T_1_ = 1220 ± 36 ms in white matter and 2132 ± 103 in cortical gray matter in adults, compared with 2933 [2893, 2972] ms and 2775 [2732, 2818] ms observed in this study; Yacoub et al.^25^ reported T_2_ = 45.9 ± 1.9 ms in white matter and 55.0 ± 4.1 ms in gray matter in adults, compared with 119 [116, 121] ms and 98 [96, 101] ms in this study. These large differences (and the reversal of contrast between white and gray matter) illustrate the importance of optimizing sequences specifically for infants. Initial results from a neonate-specific T_1_-weighted protocol optimized using these parameters were presented at a recent ISMRM meeting.^26^

Other studies have also reported age dependences in the perinatal period measured at different field strengths.^5,7–10^ Using 3T data, Schneider et al.^9^ showed a quadratic relationship between PMA and T_1_ when taken over a longer timescale (their study had many more infants aged around 30 weeks), although most tissues showed a monotonic decrease after about 35 weeks PMA, which is consistent with our data. For thalamus, for example, they showed Δ_1_ ≈ −20 ms/week, which is close to our measured value of −23 [−33, −14] ms/week. Dong et al.^23^ also reported values for 3 T, with similar values for T_1_ (e.g., Δ_1_ ≈ −28 ms/week in thalamus) but substantially larger decreases in T_2_ (e.g., Δ_2_ ≈ −3.7 ms/week in thalamus and ≈ −6.8 ms/week in white matter, compared with −1.7 [−2.0, −1.0] ms/week and −1.8 [−2.0, −1.0] ms/week, respectively, in this study). It is not clear whether this difference is due to field strength or systematic differences in the measurement techniques.

This study included infants scanned at a range of GA at birth, meaning preterm birth is an additional factor that might determine relaxation times. A second regression analysis including both PMA and PNA (Table S2) found small positive associations between postnatal age and relaxation times in some of the tissues studied; this indicates that after accounting for PMA, infants with a higher PNA (i.e., more preterm) have slightly longer relaxation times. The effect is more pronounced for T_1_ where *p*-values for association with PNA were less than 0.05 for all tissues except PVFWM. For T_2_, only white matter, basal ganglia, and brainstem showed a similar effect. To confirm our findings robustly, particularly regarding the systematic impact of prematurity, a larger number of subjects including term-born infants with a wider range of postnatal age would be needed.

### 4.1 Limitations and future work

This work focused on single-slice measurement to obtain results relatively quickly. Aside from reduced coverage, and despite careful slice positioning, not all anatomic structures of interest were found in the imaged slice for all subjects. This could contribute to the high degree of variability in measured relaxation times for smaller structures such as PVFWM. Quantitative measures were in general consistent across subjects, indicating this is not a major effect, but future work could benefit from 3D measurements.

## 5 Conclusions

We describe transverse and longitudinal relaxation times of segmented brain tissues in a cohort of neonates at 7 T. Most measured parameters correlated with PMA at scan; regression was used to determine benchmark values for term (40 weeks’ gestation) and establish the rate of change of these parameters over the perinatal period. The measured parameters may serve as a guide for future sequence optimization for studies with this age group at UHF.

## Supplementary Material

Supporting Information

## Figures and Tables

**Table 1:** Summary of linear regression analysis for T_1_ and T_2_ versus postmenstrual age. The values of $T_{1,2}^{40wk}$ are relaxation imes regressed to 40 weeks’ gestation (i.e., normal term); Δ_1,2_ is the rate of change in milliseconds per week. Both quantities are quoted along with 95% confidence intervals in brackets (*lower, upper*). *p*-Values are also quoted for each regression (the quoted *p*-value is for Δ_1,2_, where the null hypothesis is Δ_1,2_ = 0).

	T_1_				T_2_		
	$T_{1}^{40wk}(\text{ms})$	Δ_1_ (ms/week)	*p*		$T_{2}^{40wk}(\text{ms})$	Δ_2_ (ms/week)	*p*
Cortical gray matter	2775 (2732, 2818)	−12 (−17, −7)	< 0.001		98 (96, 101)	−0.9 (−1.0, −1.0)	< 0.001
White matter	2933 (2893, 2972)	−20 (−24, −17)	< 0.001		119 (116, 121)	−1.8 (−2.0, −1.0)	< 0.001
Brainstem	2347 (2292, 2401)	−11 (−24, 3)	0.109		83 (82, 84)	−1.3 (−2.0, −1.0)	< 0.001
Cerebellum	2653 (2604, 2701)	−43 (−53, −33)	< 0.001		99 (96, 102)	−4.1 (−5.0, −3.0)	< 0.001
Cerebellar vermis	2436 (2371, 2500)	−30 (−45, −14)	< 0.001		86 (83, 89)	−2.3 (−3.0, −2.0)	< 0.001
Basal ganglia	2486 (2439, 2532)	−23 (−33, −14)	< 0.001		90 (89, 92)	−2.0 (−2.0, −2.0)	< 0.001
Thalamus	2469 (2428, 2510)	−23 (−33, −14)	< 0.001		90 (89, 91)	−1.7 (−2.0, −1.0)	< 0.001
PVFWM	3165 (3107, 3222)	−24 (−35, −13)	< 0.001		146 (140, 151)	−1.3 (−2.0, −1.0)	< 0.001

Abbreviation: PVFWM, periventricular frontal white matter.

## Data Availability

Source code and data required to reproduce the results from this paper and supporting figures are available online at https://github.com/mriphysics/7T-neonate-t1-t2-mapping (most recent commit hash 3bbee74). This includes image data and fitting code for phantom experiments and one neonate example, fitted parameter maps from all neonates, and scripts to replicate all statistical analysis.
