# Supplementary material for: T1 and T2 measurements of the neonatal brain at 7 T
Source: Magn Reson Med. 2024 Dec 13;93(5):2153–62. doi: 10.1002/mrm.30403 (PMC7617262; doi:10.1002/mrm.30403)
Supplement: Supplementary file 1 — Figure S1. Left column: Raw simulated signals for the three echo times. Right column: Interpolated values used for dictionary estimation. Figure S2. Bias resulting from assuming fixed T1. This was estimated by randomly sampling from a full dictionary including T1 variation, then estimating T2 by assuming fixed T1 = 2.6 s. Bias remains < 2% for 2 s < T1 < 3.5 s for B1rel>0.5, which covers most of the expected conditions for neonatal imaging (see Figure 2C). Figure S3. Inversion recovery–based T1 estimation, fitting with and without parameter ϵ in Eq. (1). Top panel: Results for the standard version of the sequence, using 129 V for the inversion pulse; there is no obvious dependence of estimated T1 on B1+ either with or without ϵ. Bottom panel: The inversion‐pulse voltage was intentionally reduced to cause incomplete inversion in areas of low B1+. In this case, when ϵ is not included in fitting, the estimated T1 has strong B1+ dependence, but this is cleared up by including ϵ in fitting. Figure S4. Phantom validation of T2 measurement. Top left: Single slice from three‐dimensional (3D) spin‐echo measurement (the holes are caused by coil combination error in the image reconstruction). Top middle image: T2 estimated from exponential fit to two‐dimensional (2D) turbo spin echo (TSE)–based measurement. Top right: Reconstruction of the same 2D TSE data using dictionary reconstruction. Bottom left panel: B1rel map for reference. Bottom right panel: Histograms for all methods. Estimated T2 values from each method are 3D spin echo (SE) = 107.5 ± 2.4 ms, two‐dimensional (2D) without correction = 154.5 ± 7.1 ms, and 2D with correction = 111.4 ± 5.3 ms. Figure S5. T1 maps from 40 subjects. Note that the subjects are ordered by increasing postmenstrual age. Figure S6. Inversion inefficiency parameter maps from T1 estimation in all subjects. Figure S7. T2 maps from all subjects where estimation was possible (i.e., where B1 information was also obtained). Figure S8. Region‐of‐inter [file MRM-93-2153-s001.pdf]

Supporting Information

ID #	sex	GA	PMA	B1 MAP?	Rescan	Scan rejected (ms)	DIAGNOSIS
2	F	41 +1	43 + 2	N	NO	NO	Maternal history of autism, otherwise well
3	F	38 + 3	43 + 4	Y	NO	NO	Congenital CMV infection, marked polymicrogyria, periventricular cysts, white matter hyperintensity, calcification
4	M	31 + 5	44 + 2	N	NO	NO	Preterm, healthy brain
5	M	38 + 4	48	N	NO	No IR	Hypoxic ischaemic encephalopathy, neonatal seizures
6	F	38 + 5	40 + 3	N	NO	NO	Antenatal ventriculomegaly, maternal mental health difficulties, consanguineous parents. Low tone and feeding difficulties
7	M	37 + 3	37 + 6	N	#18	500, 1000	Antenatal TGA and VSD
8	M	40 + 3	41	N	NO	NO	Meconium aspiration
9	M	34 + 3	35 + 6	Y	NO	NO	Preterm, emergency LSCS for breach presentation. CPAP for 2 days
10	M	33 + 1	38 + 5	Y	NO	NO	Preterm due to maternal chorioamnionitis, CPAP for 1 day, 5 days antibiotics for possible sepsis. Small white matter cyst seen on 3T scan.
11	M	39 + 3	40 + 3	Y	NO	NO	Antenatal diagnosis TGA - pre-op
12	M	39 + 5	41 + 1	Y	NO	NO	Antenatal diagnosis TGA - post-op, arterial switch 28/11/2022
13	M	34	35 + 6	Y	#14	5000	Preterm, absent CSP, transverse lie + cord prolapse
14	M	34	39 + 5	Y	#13	NO	Preterm, absent CSP, transverse lie + cord prolapse
15	F	39 + 6	41 + 6	Y	NO	NO	Maternal group a strep, oral antibiotics
16	M	40 + 2	41 + 2	Y	NO	5000	Partial anomalous pulmonary venous drainage and possible coarctation
17	M	34 + 6	37 + 5	Y	NO	NO	Suspected coarctation, left sinus hypoplasia
18	M	37 + 3	52 + 6	N	#7	1500, 2000, 3000, 4000, 5000	Post-op TGA and VSD
19	M	41 + 4	44 + 5	N	NO	NO	Term baby, forceps and ventouse
20	M	27 + 6	34 + 3	Y	#27	NO	Preterm infant

21	M	34 + 2	34 + 5	Y	#28	NO	Preterm infant, bilateral subependymal cysts
22	M	38 + 4	40	Y	NO	NO	Agenesis of the corpus callosum, ventriculomegaly
23	F	37 + 5	40 + 1	Y	NO	NO	Pulmonary stenosis, tetralogy of fallot
24	F	40	40 + 2	Y	NO	4000	Antenatal diagnosed conatal cysts
25	F	34 + 3	36 + 4	Y	#26	NO	Preterm infant, cholestasis
26	F	34 + 3	41 + 4	Y	#25	NO	Preterm infant, cholestasis
27	M	27 + 6	42 + 3	Y	#20	5000	Preterm infant (follow-up)
28	M	34 + 2	42 + 4	Y	#21	NO	Preterm infant (follow-up)
29	M	28 + 4	39 + 5	Y	NO	NO	preterm infant, PROM, chronic lung disease, punctate lesions
30	F	38	39 + 4	Y	NO	NO	Partial agensis of the corpus callosum, imperforate hymen
32	M	33	35 + 1	Y	NO	NO	Preterm, maternal chorioamnionitis
33	F	34 + 1	36 + 5	Y	NO	NO	Preterm, fetal distress, fetal-maternal haemorrhage
37	M	31 + 2	33 + 4	Y	NO	NO	Preterm, spontaneous preterm labour
42	M	35	42 + 6	Y	#35	NO	Preterm, maternal sickle cell trait
49	M	31 + 2	38	Y	NO	4000	Preterm, DCDA twin IVF, gestational diabetes
50	M	31+ 2	38	Y	#51	NO	Preterm, DCDA twin IVF, gestational diabetes
51	M	31 + 1	38 + 4	Y	#50	NO	Preterm, hypoglycaemia, IUGR
52	F	35 + 3	36 + 5	Y	NO	NO	Preterm, VSD
53	M	35 + 1	37	Y	NO	4000, 5000	Preterm, previous maternal preterm births
54	F	36 + 3	36 + 6	Y	NO	NO	Late preterm
55	F	31 + 2	35	Y	NO	NO	Preterm maternal chorioamnionitis, cerebral haemorrhage, history of sepsis

Supporting Information Table S1: Patient demographic and diagnostic details including: gender; PMA (post-menstrual age at scan, weeks+days); GA (gestational age at birth, weeks+days); B1 map acquisition status for T2 correction (yes or no); repeat scans (either no, or the associated scan number for the same infant); rejected images T1 mapping (if not NO then numbers are the Ti of rejected scans in ms); and clinical information.

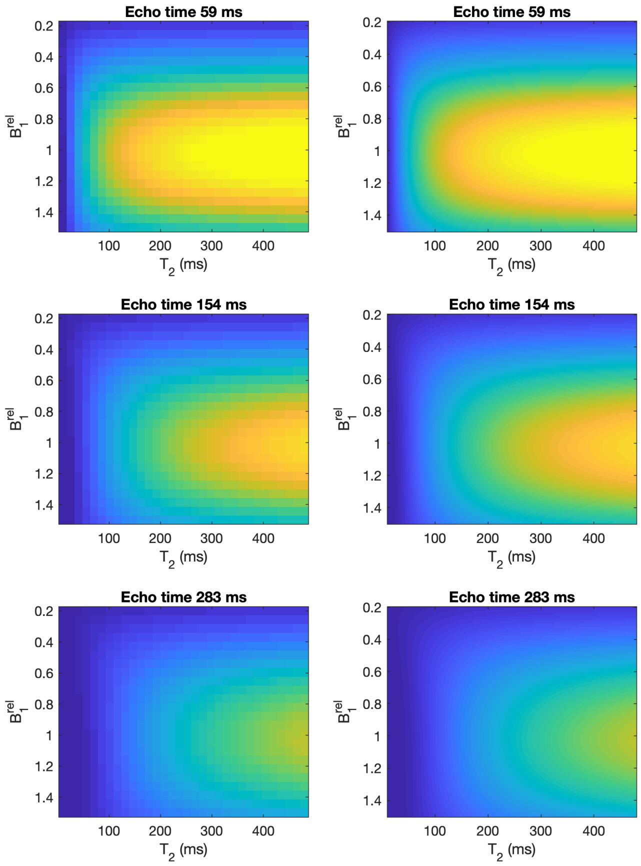

Supporting Information Figure S1: Left column, raw simulated signals for the 3 echo times; Right column, interpolated values used for dictionary estimation.

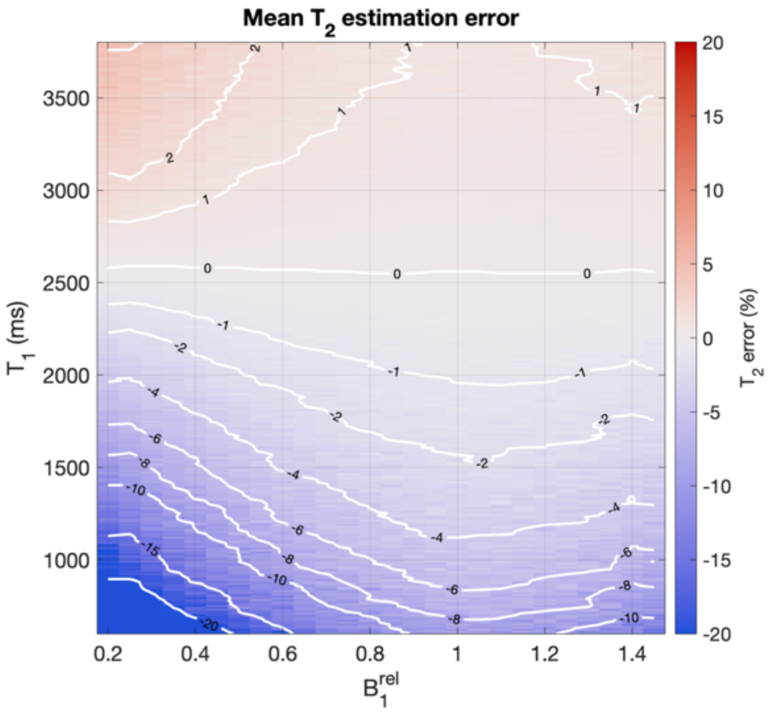

Supporting Information Figure S2: Bias resulting from assuming fixed T_1. This was estimated by randomly sampling from a full dictionary including T_1 variation, then estimating T_2 by assuming fixed T_1=2.6s. Bias remains <2% for 2s<T_1<3.5s for $B_1^{rel} > 0.5$ which covers most of the expected conditions for neonatal imaging (e.g. see Figure 2c).

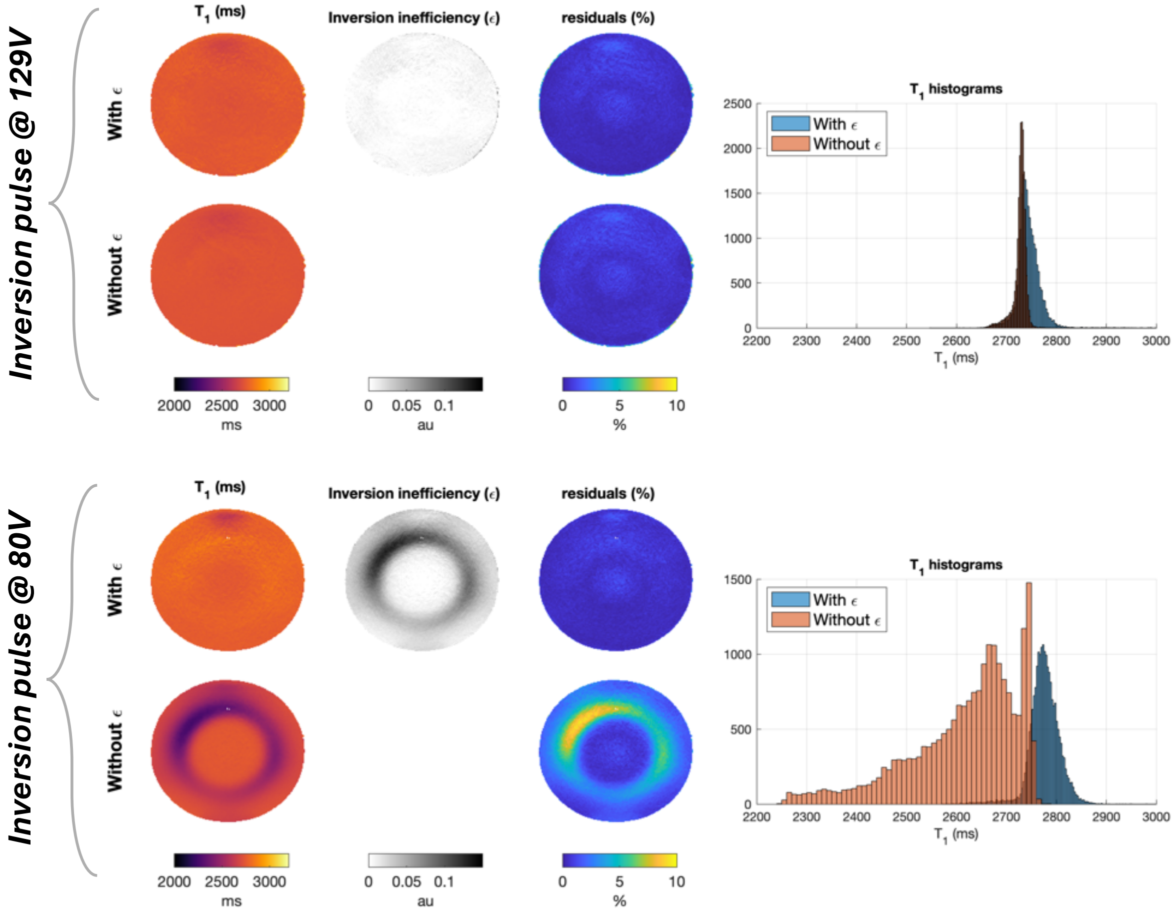

Supporting Information Figure S3: Inversion recovery based T1 estimation, fitting with and without parameter ϵ in Eq.[1]. Top panel: results for the standard version of the sequence, using 129V for the inversion pulse – there is no obvious dependence of estimated T_1 on B_1^+ either with or without ϵ . Bottom panel: The inversion pulse voltage was intentionally reduced to cause incomplete inversion in areas of low B_1^+. In this case when ϵ is not included in fitting, the estimated T_1 has strong B_1^+ dependence, but this is cleared up by including ϵ in fitting.

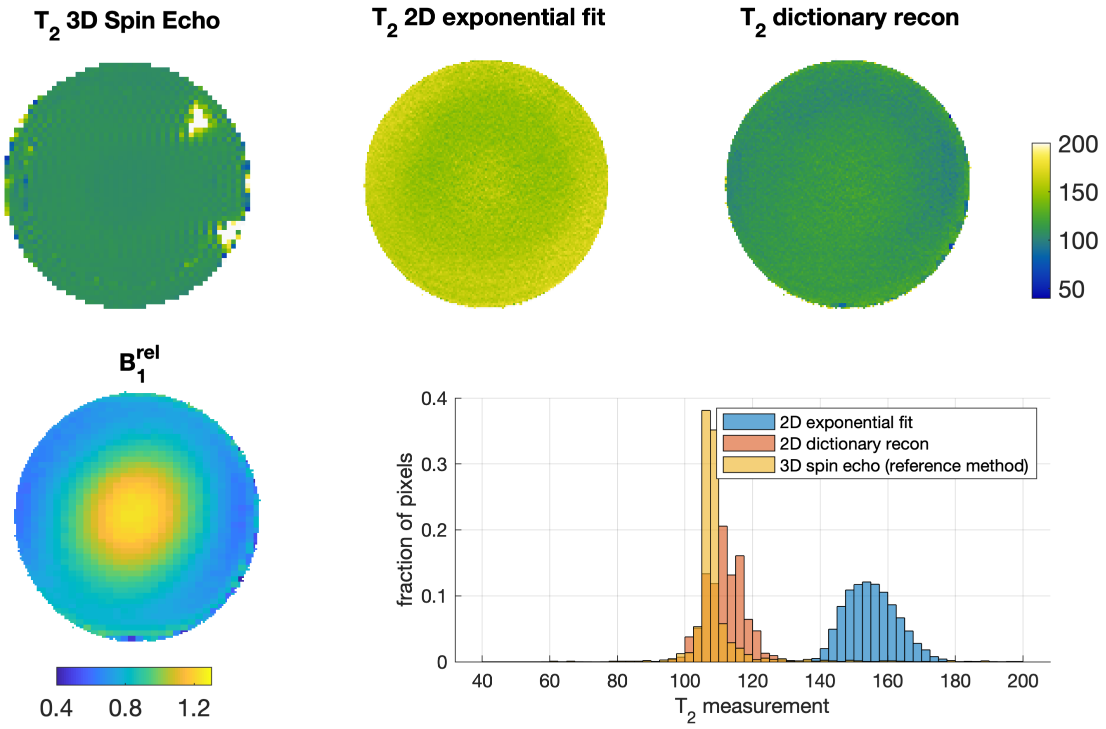

Supporting Information Figure S4: Phantom validation of T2 measurement. Top left image shows single slice from 3D spin echo measurement (the holes are caused by coil combination error in the image reconstruction); top middle image is T2 estimated from exponential fit to 2D TSE based measurement; top right is reconstruction of the same 2D TSE data using dictionary reconstruction. Bottom left panel shows B_1^{rel} map for reference. Bottom right shows histograms for all methods. Estimated T2 values from each method are: 3D SE 107.5±2.4ms, 2D without correction 154.5±7.1ms, 2D with correction 111.4±5.3ms.

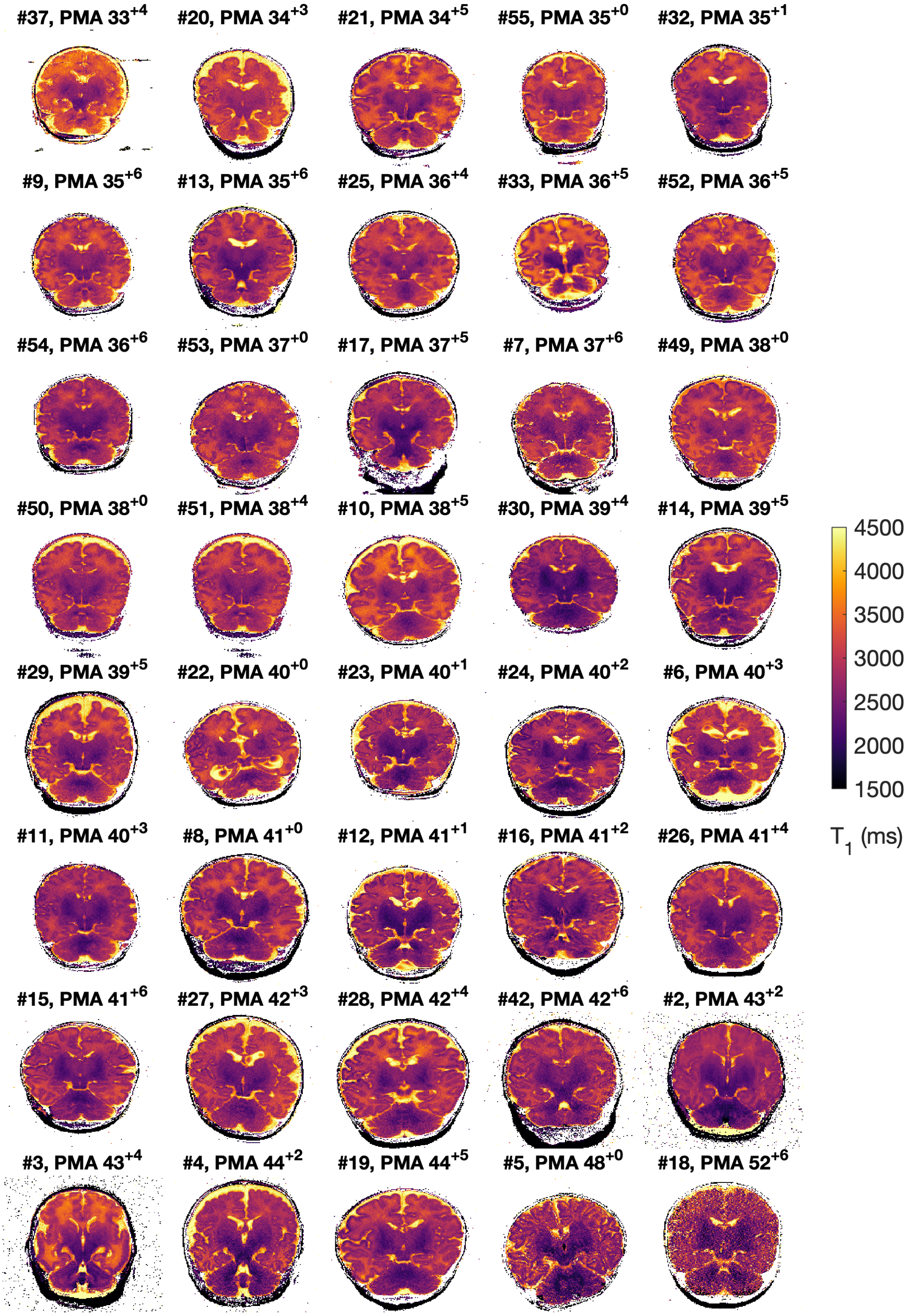

Supporting Information Figure S5: T1 maps from 40 subjects. Note that the subjects are ordered by increasing post menstrual age.

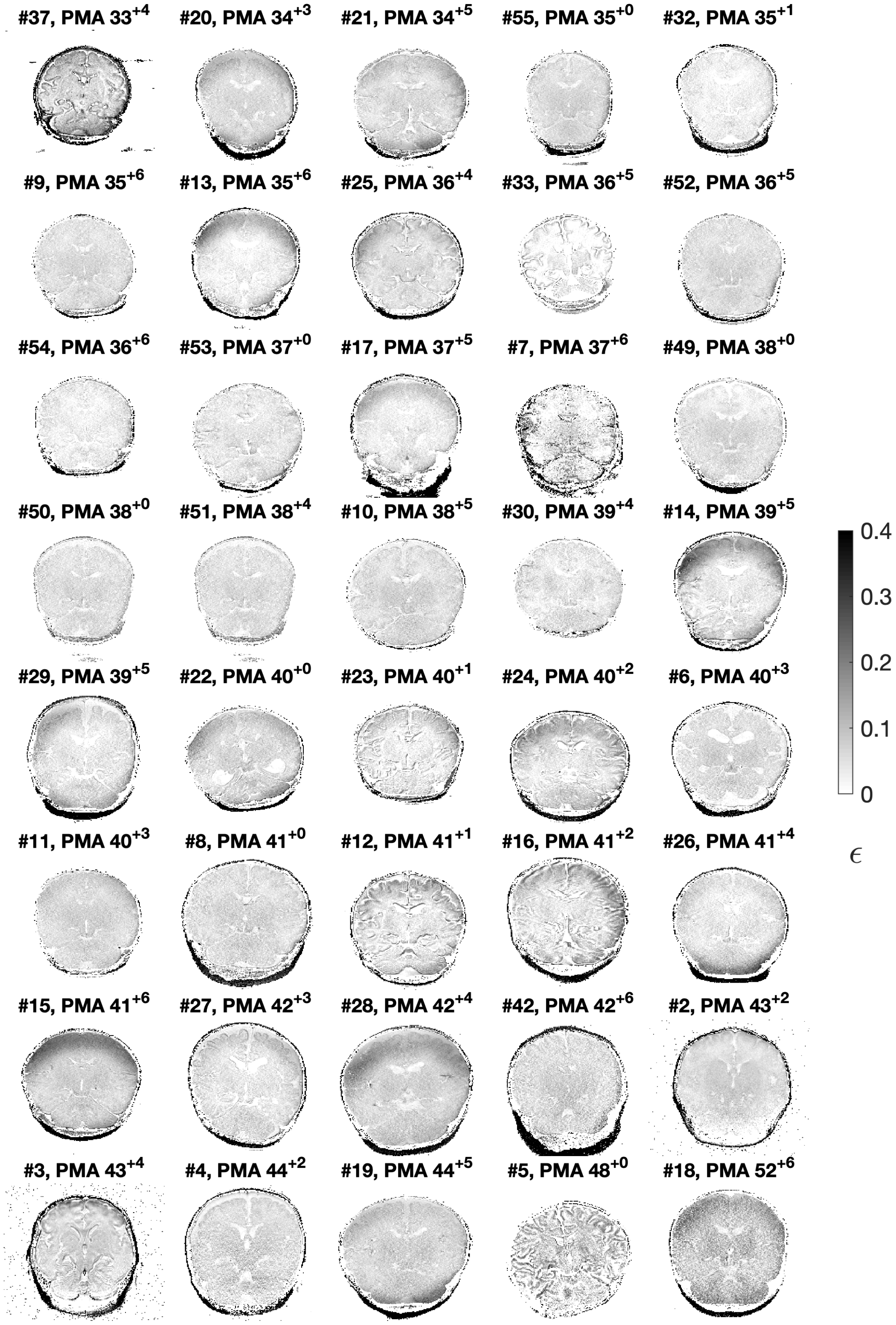

Supporting Information Figure S6: Inversion inefficiency parameter maps from T1 estimation in all subjects

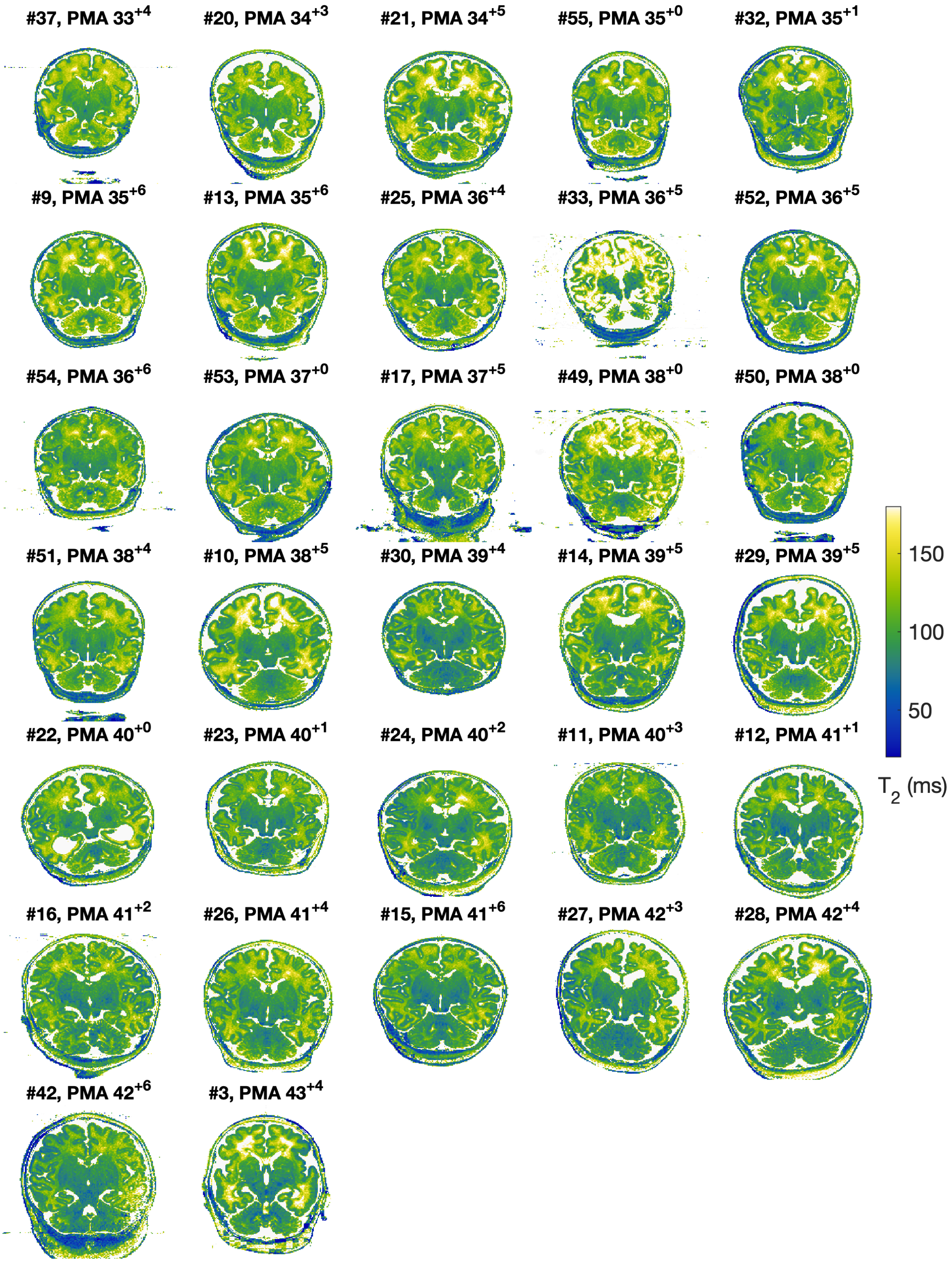

Supporting Information Figure S7: T2 maps from all subjects where estimation was possible (i.e. where B1 information was also obtained).

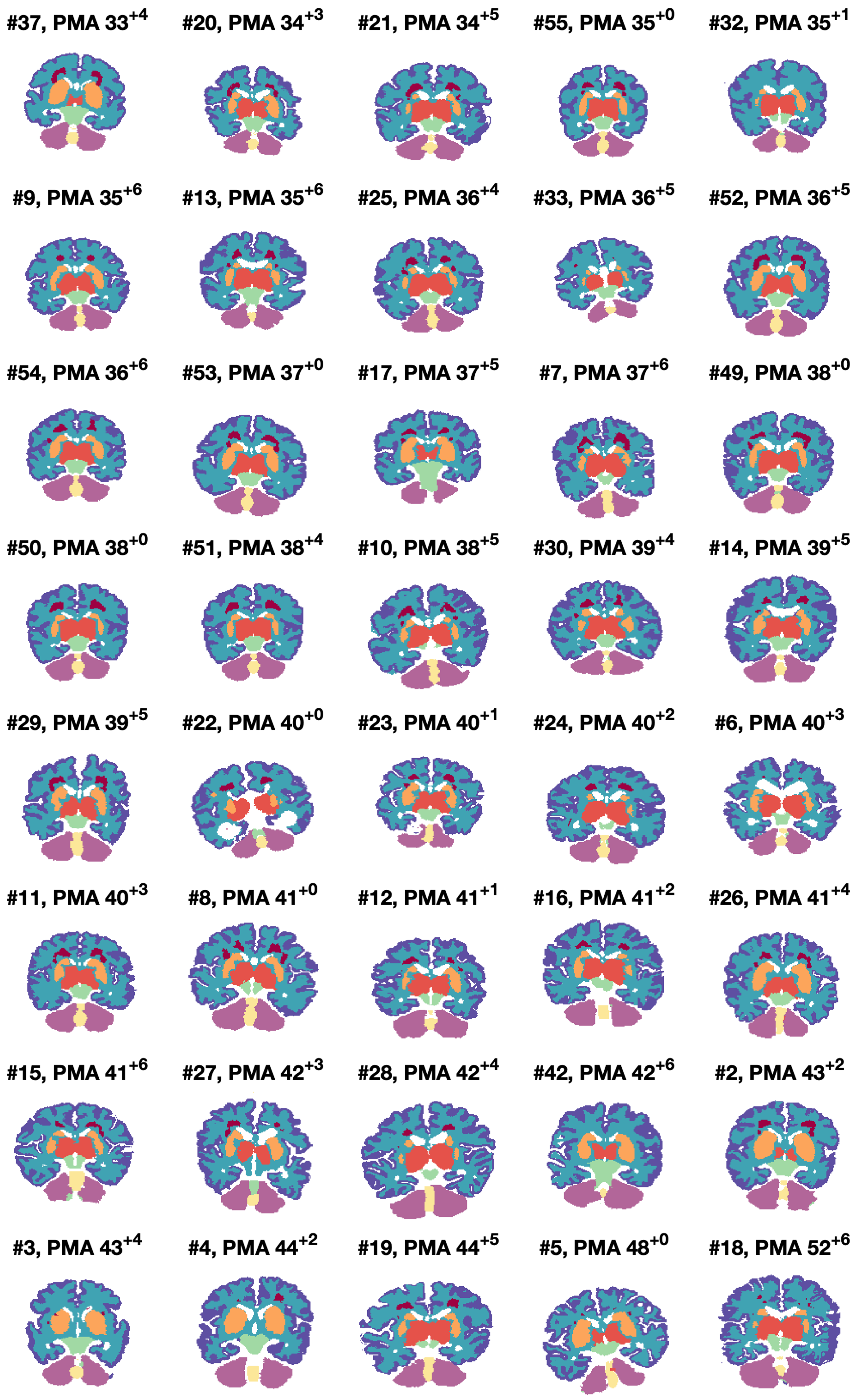

Supporting Information Figure S8: ROI labels for all data (color key is given in Figure 1). Some structures such as brain stem and thalamus are not present in all subjects because of small differences in positioning and anatomical variation of these structures.

Result of multiple linear regression

	T1					T2				
	T_1^{40wk} (ms)	Δ_1^{PMA} (ms/wk)	p	Δ_1^{PNA} (ms/wk)	p	T_2^{40wk} (ms)	Δ_2^{PMA} (ms/wk)	p	Δ_2^{PNA} (ms/wk)	p
Cortical GM	2710 (2651,2769)	-26 (-37,-15)	< 0.001	15 (5,26)	0.006	97 (93,101)	-1.2 (-2.0,-0.0)	0.002	0.3 (-0.0,1.0)	0.414
White Matter	2882 (2825,2938)	-32 (-42,-22)	< 0.001	12 (2,22)	0.021	115 (111,119)	-2.3 (-3.0,-2.0)	< 0.001	0.8 (0.0,1.0)	0.018
Brainstem	2272 (2191,2353)	-22 (-38,-6)	0.008	17 (3,32)	0.021	81 (79,83)	-1.4 (-2.0,-1.0)	< 0.001	0.4 (0.0,1.0)	0.017
Cerebellum	2575 (2506,2643)	-57 (-70,-44)	< 0.001	18 (6,30)	0.005	99 (95,103)	-4.1 (-5.0,-3.0)	< 0.001	0.1 (-1.0,1.0)	0.875
Cerebellar Vermis	2314 (2230,2398)	-48 (-64,-32)	< 0.001	28 (13,43)	< 0.001	84 (80,89)	-2.6 (-3.0,-2.0)	< 0.001	0.5 (-0.0,1.0)	0.231
Basal Ganglia	2389 (2333,2446)	-40 (-51,-29)	< 0.001	23 (12,33)	< 0.001	88 (86,90)	-2.4 (-3.0,-2.0)	< 0.001	0.6 (0.0,1.0)	0.001
Thalamus	2423 (2363,2483)	-28 (-40,-16)	< 0.001	12 (1,22)	0.040	89 (87,91)	-1.8 (-2.0,-1.0)	< 0.001	0.2 (-0.0,1.0)	0.156
PVFWM	3115 (3029,3201)	-34 (-50,-17)	< 0.001	12 (-4,27)	0.126	141 (132,150)	-2.2 (-4.0,-1.0)	0.009	1.0 (-1.0,3.0)	0.221

Supporting Information Table S2: Results of mixed model $T_{1,2} = T_{1,2}^{40wk} + (PMA - 40) \times \Delta_{1,2}^{PMA} + PNA \times \Delta_{1,2}^{PNA} + (1|subject\_id)$. Here, PNA is 'postnatal age' which is defined as PMA-GA – i.e. it is the time in weeks since birth. The p-value to the right of each Δ coefficient is the value for that specific coefficient, with null hypothesis that it is zero.
